# CD147 and matrix-metalloproteinase-2 expression in metastatic and non-metastatic uveal melanomas

**DOI:** 10.1186/s12886-016-0222-4

**Published:** 2016-06-03

**Authors:** Julia Lüke, Vlatka Vukoja, Tim Brandenbusch, Khaled Nassar, Jens Martin Rohrbach, Salvatore Grisanti, Matthias Lüke, Aysegül Tura

**Affiliations:** Department of Ophthalmology, University of Lübeck, Ratzeburger Allee 160, 23538 Lübeck, Germany; University Eye Hospital, Centre of Ophthalmology, Eberhard-Karls University of Tuebingen, Tuebingen, Germany

## Abstract

**Background:**

Extracellular matrix remodelling regulated by matrix-metalloproteinase (MMP) inducer (CD147) is a crucial process during tumor cell invasion and regulation of blood supply. In this study, we evaluated the correlation of CD147 and MMP-2 expression with major prognostic factors for uveal melanoma and the development of metastasis.

**Methods:**

The expression of CD147 and MMP-2 was analyzed in 49 samples of uveal melanomas. Triple immunofluorescence stainings using markers against glial cells (GFAP), endothelial cells (CD34) and macrophages (CD68) were performed to further analyse the exact localisation of CD147 and MMP-2 positivity. In 28 cases clinical metastatic disease were found. The remaining 21 cases showed no signs of metastatic disease for an average follow-up of 10 years. Correlation analysis (Pearson correlation) was performed to analyse the association of CD147 and MMP-2 expression with known prognostic factors, vasculogenic mimicry *(VM)*, the mature vasculature (von Willebrand Factor) and tumor induced angiogenesis (by means of Endoglin expression).

**Results:**

CD147 and MMP-2 were expressed in 47 (96.0 %) of the uveal melanomas. CD147 up-regulation was significantly correlated with a higher MMP-2 expression. The overall expression analysis revealed no significant difference in the metastatic (*p =* 0.777) and non-metastatic subgroup (*p =* 0.585). No correlation of CD147 expression and any system of blood supply was evident. In the non-metastatic sub-group a significant correlation of clustered CD147 positive cells with largest basal diameter (*p =* 0.039), height (*p =* 0.047) and TNM-stage (*p =* 0.013) was evident.

**Conclusions:**

These data may indicate that CD147 regulates MMP-2 expression in uveal melanoma cells.

## Background

Uveal melanoma is the most frequent primary intraocular malignancy in Caucasians. Tumor growth is dependent on the blood supply, which is delivered on the one hand by angiogenesis. Angiogenesis includes the outgrowth and proliferation of endothelial cells that remodel the extracellular matrix and align into tube-like structures, and eventually form functional blood vessels. On the other hand a supplementary blood supply is present in uveal melanomas. Maniotis et al. first described the phenomenon that highly aggressive and metastatic melanoma cells form vascular channels lined externally by tumor cells, which are attached to a basement membrane without the presence of endothelial cells [1]. This process was termed *vasculogenic mimicry (VM)*. This seems to play a pivotal role as an alternative pathway when conventional angiogenesis is inhibited.

During angiogenesis and vasculogenic mimicry extracellular matrix remodelling is a crucial process. It involves numerous extracellular matrices – degrading enzymes including matrix metalloproteinases (MMPs), which are believed not only to affect tumor angiogenesis and *VM* but also tumor growth, local invasion, and subsequent distant metastasis [2, 3]. MMPs are metal-dependent endopeptidases sharing a common modular domain structure. They cleave the extracellular matrix components of the parenchymal and vascular basement membranes which are normally mechanical barriers to cell migration and invasion. In tumor local environments MMPs are usually overproduced; an overexpression of MMPs have been correlated with tumor vascular density [2, 3]. In an in vivo tumor model, an increased MMP activity was observed in association with tumor angiogenic potential [4, 5].

MMP stimulation is partly regulated by the extracellular MMP inducer (EMMPRIN/CD147), a membrane glycoprotein greatly enriched on the surface of tumor cells that is known to stimulate tumor and neighbouring stromal cells, such as fibroblasts and endothelial cells, to increase their synthesis of several MMPs [6–10]. Previous studies showed that CD147 is present in the corneal epithelium, stromal keratocytes, endothelial cells, Bowman membrane in keratoconic corneas, in the RPE, different retinal layers, and nerve fascicles in the optic nerve head [11–13]. CD147 expression has been correlated with invasion and tumor progression in numerous malignant tumor models including melanoma [14, 15].

So far the CD147 expression and their association with established prognostic factors, with different modalities of blood supply as well as the associated target molecule MMP-2, have not been studied in uveal melanomas. Therefore, to investigate the CD147 and MMP-2 signalling pathway we evaluated their expression and correlation with known prognostic factors, *VM*, the mature vasculature and angiogenesis in metastatic and non-metastatic uveal melanomas.

## Methods

### Immunohistochemistry

Paraffin sections from 49 of primary uveal melanoma, which were pathologically well characterized (See Table 1), were analyzed retrospectively with respect to the immunohistological expression of CD147 and MMP2. Staining for von Willebrand Factor (vWF), a marker for mature vasculature, was performed to allow the count of vessels. For visualisation of tumor induced angiogenesis, immunohistochemical staining for Endoglin (CD105) was carried out. During neoplasm-related angiogenesis the transforming growth factor-β1 and –β3 (TGF- β1, TGF-β3) binding receptor Endoglin is strongly up-regulated on the endothelium [16, 17]. All the primary and secondary antibodies used are listed in Table 2, with the information on their type, source, and dilution factor, as well as the corresponding pretreatment and visualisation methods.

**Table 1:** Clinical data of the patients and histological characteristics of the uveal melanomas

Variable	Total	Metastasis	No metastasis	P-Wert (Spearman’s Rho Test)	
				*p*-value	*r*-value
Number of patients	49	28	21		
Sex					
Female	21 (42.9 %)	13 (26.5 %)	8 (16.3 %)	0.894	−0.019
Male	28 (57.1 %)	15 (30.6 %)	13 (26.5 %)		
LTD (mm)					
Median	15^a^	15.5^b^	15^b^	0.164	0.208
* Range*	*5–30*	*10–30*	*5–28*		
Height (mm)					
Median	7^c^	7.5^a^	6^b^	0.263	0.175
Range	2–22	2-	2–12		
Ciliary body invasion	22^d^ (44.9 %)	16 (57.1 %)	6^d^ (28.6 *%*)	0.108	0.230
Scleral invasion	46^d^ (93.9 %)	26 (92.9 %)	20^d^ (95.2 %)	0.247	−0.149
Extrascleral extension	7^e^ (14.3 %)	5^d^ (17.9 %)	2^d^ (9.5 %)	0.058	−0.282
Invasion					
of Bruch’s membrane	27^d^ (55.1 %)	16 (57.1 %)	11^d^ (52.4 %)	0.732	0.050
of the optic nerve head	2^d^ (4.1 %)	1 (3.6 %)	1^d^ (4.7 %)	0.560	−0.088
Histological subtype					
Epithelioid	9 (18.4 %)	8 (28.6 %)	0 (0 %)	0.005	0.387
Mixed	22 (44.9 %)	12 (42.9 %)	11 (52.4 %)		
Spindle cell	18 (36.7 %)	8 (28.6 %)	10 (47.6 %)		
TNM					
Stage I	5^f^ (10.9 %)	0^d^ (0.0 %)	5^c^ (26.3 %)	0.073	0.264
Stage II	10 (21.7 %)	6 (22.2 %)	4 (21.1 %)		
Stage III	16 (34.8 %)	11 (40.7 %)	5 (26.3 %)		
Stage IV	15 (32.6 %)	10 (37.0 %)	5 (26.3 %)		
High CD105 expression	26^d^ (54.2 %)	18^d^ (66.7 %)	8 (38.1 %)	0.098	0.237
High vWF expression	26 (53.1 %)	18 (64.3 %)	8 (21.1 %)	0.129	0.216
VM	25 (51.0 *%*)	19 (67. *%*)	6 (28.6 %)	0.016	0.342

*LTD* largest tumor diameter, *mm* Millimeter, *TNM* Tumor Node Metastasis, *CD105* Endoglin, *vWF* von Willebrand Factor, *VM Vasculogenic Mimicry*

^a^4 missing values, ^b^2 missing values, ^c^6 missing values, ^d^1 missing value, ^e^2 missing values, ^f^3 missing values

**Table 2:** Summary of the applied primary and secondary antibodies

Primary antibodies						
Antigen	Source	Dilution	Clone	Manufacturer	Pre-treatment	Visualisation
CD147	Mouse	1:50	P	Abcam, Cambridge, UK	Cooked in citrate puffer	Fluorescence (Alexa Fluor® 488)
MMP-2	Rabbit	1:100	P	Abcam, Cambridge, UK	Cooked in citrate puffer	Fluorescence (Cy3)
CD34	Rat	1:100	Clone MEC 14.7	Abcam, Cambridge, UK	Cooked in citrate puffer	Fluorescence (Cy5)
CD68	Rat	1:100	Clone FA-11	Abcam, Cambridge, UK	Cooked in citrate puffer	Fluorescence (Cy5)
GFAP	Chicken	1:1000	P	Abcam, Cambridge, UK	Cooked in citrate puffer	Fluorescence (Alexa Fluor® 647)
Endoglin	Mouse	1:5	Clone SN66	Dako, Glostrup, Denmark	100 mg Protease in 200 ml TBS	Chromogenic (HRP, DAB)
vWF	Mouse	1:50	Clone F8/86	Dako, Glostrup, Denmark	100 mg Protease in 200 ml TBS	Chromogenic (HRP, DAB)
CD31	Mouse	1:40	Clone JC704	Dako, Glostrup, Denmark	Cooked in TBS	Chromogenic (HRP, DAB)
Secondary antibodies						
Reactivity	Source	Dilution	Clone	Conjugation	Manufacturer	
Mouse IgG (H + L)	Goat	1:50	P	Alexa Fluor® 488	Molecular Probes®- Invitrogen, Eugene,USA	
Rabbit IgG (H + L)	Goat	1:50	P	Cy3	Dianova, Hamburg, Germany	
Rat IgG (H + L)	Donkey	1:50	P	Cy5	Dianova, Hamburg, Germany	
Chicken IgY (H + L)	Goat	1:50	P	Alexa Fluor® 647	Dianova, Hamburg, Germany	
Mouse IgG	Goat	1:250	P	HRP	Dianova, Hamburg, Germany	

*DAB* 3,3′-Diaminobenzidine, *HRP* Horseradish peroxidase, *P* Polyclonal, *TBS* Tris-buffered saline

The uveal melanomas were obtained by enucleation from 1982 to 1999 at the University Eye Hospital of Tübingen (specimens’ use authorized by JMR, head of ophthalmopathology at the University Eye Hospital of Tübingen). In 28 cases metastatic disease was found in the restaging examination. The remaining 21 patients had a mean follow-up of 10 (6–18) years. Irradiation was conducted previously in 16 tumors. The non irradiated tumors received enucleation as primary therapy.

For CD147 and MMP-2 double-immunostainings, serial paraffin sections were deparaffinized in three changes of xylol and rehydrated in a graded series of alcohol (100–50 %). Antigen retrieval was performed by cooking the slides in citrate puffer for 25 min, followed by cooling down at room temperature for 20 min and washing three times with phosphate-buffered saline (PBS). Afterwards, sections were incubated with blocking buffer (2 % bovine serum albumin (w/v) and 20 % rat serum (v/v) in PBS) for one hour at room temperature followed by the coincubation with primary CD147 and MMP-2 antibodies (diluted as stated in Table 2 in 3%BSA-0.1 % Triton X-100 in PBS) overnight at 4 °C. Negative controls were incubated in the blocking buffer alone. Sections of mamma carcinoma were incubated with the primary antibody cocktail as positive control. After rinsing with PBS, sections were incubated with the corresponding fluorescence-conjugated secondary antibodies (Table 2) diluted in 3 % BSA-PBS for 1 h at room temperature under protection from light. After rinsing with PBS three times for 5 min, nuclei were counterstained with DAPI (0.5 μg/ml in PBS, 150 μl per section) for 8 min in the dark. After rinsing with PBS for 5 min, the slides were mounted with Mowiol-coated coverslips.

Triple fluorescence-immunostainings were performed by following the procedure above, by incubating the sections with a cocktail of primary antibodies against CD147, MMP-2 and CD34, CD68 or GFAP, followed by the cocktail of secondary antibodies, which were diluted in 3 %BSA-0.1 % Triton X-100 in PBS as described in Table 2.

The Endoglin and vWF immunostainings were performed as described previously [18]. In brief, the sections were deparaffinized and rehydrated as above. In order to better visualize the staining, melanin was bleached by incubating the sections in 3.0 % (v/v) hydrogen peroxide, 0.5 % potassium hydrogen phosphate, and 1.0 % (w/v) disodium hydrogen phosphate for 12 h at room temperature as described previously [19]. The majority (*n =* 26) of slides received a bleaching due to the high level of pigmentation with ranging bleaching times (mean: 65.2 min) depending on the pigmentation grade. No background staining arising from endogenous peroxidases was detected in the negative controls of the remaining unbleached slides (data not shown). Antigen retrieval by proteolytic digestion was performed on the sections that would be processed for Endoglin immunostaining by incubation in 0.5 % Pronase (Sigma-Aldrich, Munich, Germany) in PBS for 30 min. Sections were incubated with horse serum (15 μl in 1 ml PBS) for 30 min at room temperature, followed by the primary antibodies against human Endoglin or vWF (Table 2) for 12 h at 4 °C. Appropriate normal serum instead of the primary antibodies was added for negative controls. Paraffin sections of eyes that have shown a high positivity in a previous staining served as positive controls. After three washes for 5 min in PBS, sections were incubated with horseradish peroxidase-conjugated secondary antibodies for 1 h at room temperature. Before counterstaining with Mayer’s hematoxylin and coverslipping the slides were washed in PBS and developed with 3-Diaminobenzidine (Fluka, Buchs, Switzerland).

To visualize *VM* CD31/PAS-immunostaining was performed as previously described [18]. In brief, sections were deparaffinized and bleached as above. Antigen retrieval was performed by cooking the sections in Tris-buffered saline (TBS) for 30 min. After blocking with horse serum (15 μl in 1 ml TBS) for 60 min, sections were incubated with the primary antibodies against CD31, followed by the horseradish peroxidase-conjugated secondary antibodies (Table 2) for 1 h at room temperature each. After three rinses for 5 min in PBS, sections were developed with 3-Diaminobenzidine (Fluka, Buchs, Switzerland), counterstained with periodic acid-Schiff, and coverslipped. Negative controls of the tumor sections and gastric mucosa were treated with normal sera instead of antibodies and all exhibited a negative staining (data not shown).

### Image analysis

Three days after immunohistochemistry, images were acquired using a fluorescence microscope (Leica DMI 6000B, Solms, Germany connected to a digital camera (Leica DFC 290, Solms, Germany) and the corresponding filters (A4: Ex: 360/40, Em: 470/40 nm; L5: Ex: 460/40, Em: 527/30 nm; Y3: Ex: 545/30, Em: 610/75 nm; Y5: Ex: 620/60, Em: 700/75 nm). All tumor samples were initially observed under 100X magnification to evaluate the homogeneity of CD147 and MMP-2 stainings. For the quantification of CD147 and MMP-2 expression, images from *n =* 10 random fields of 0.04 mm^2^ (400X magnification) were acquired, ensuring that the images are representative of the areas exhibiting different intensities in the case of tumors having inhomogeneous antigen expression. Quantifications were performed using the ImageJ Software (National Institute of Mental Health, Bethesda, Maryland, USA) by two independent observers (JL, VV), who were totally masked to the follow-up of the patients. Evaluation of the antigen expression in tumor samples was conducted in 10 random fields of 0.04 mm^2^ (400X magnification) with the ImageJ Software (National Institute of Mental Health, Bethesda, Maryland, USA) by two independent observers (JL, VV) who were totally masked to the follow-up of the patients. Additionally, the presence of clustered CD147 positive cells was evaluated: (no: 0, moderate number: 1, or high number: 2).

The objective evaluation of the fluorescence intensity was performed using the ImageJ software. Ten images were summarized as image stack and a grid was placed over the pictures with an edge length of 3.5 in. resulting in 680 rectangles. A rectangle was counted as positive if 50 % of the area was positive. The intensity of the pixels in stack was calculated according to the formula: V = (R + G + B)/3. At the end of the calculation, the percentage mean intensity was calculated from the mean of the intensity of stained areas for each marker. The intensity was then classified: (no expression: no positive cells (0), low intensity (1): 1–24 %, moderate intensity (2): 25–50 %, high intensity (3): 51–75 %, very high intensity (4): 76–100 %). The following formula was used for calculating the objective expression rate: 100/16 x cell count x intensity score.

To evaluate the status of mature vasculature and angiogenesis the number of all vWF and Endoglin positive cells was evaluated in *n =* 3 of the 10 images (0.04 mm^2^) exhibiting the highest level of expression according to the following grading system: no expression: no positive cells, low/moderate expression: 1–50 %, high expression: 51–100 %) as reported previously [18].

**Fig. 1:**
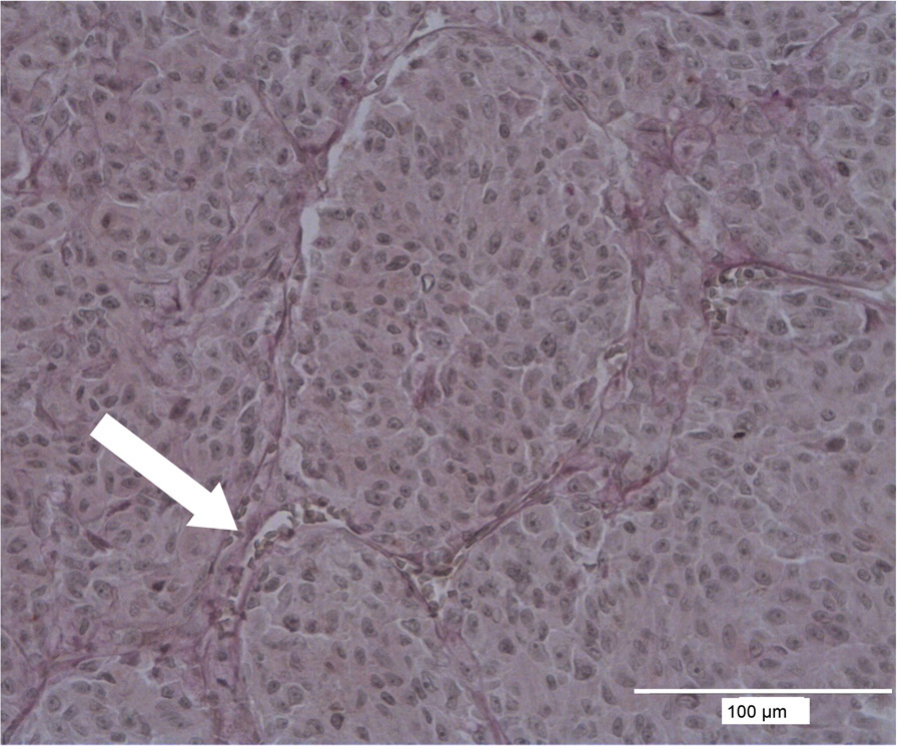
Immunofluorescence microphotography of CD31/PAS-staining demonstrating a VM typical loop. Part of a PAS positive channel containing red blood cells is marked with an arrow

The analysis of *VM* two independent observers (JL, VV) analyzed the CD31 and PAS stained uveal melanoma specimens under 200x and 400x magnification (Leica DMI 6000B, Solms, Germany) and assigned the tumors to be VM positive or VM negative. A positive rating was given in case of vascular channel formation as reported previously [1]. An example is shown in Fig. 1. Sections with different rates by first and second observer were re-evaluated to come to a final decision.

### Statistics

The data were described by means and standard deviation (±SD). A Mann–Whitney-*U* test and a correlation analysis (Pearson correlation) were performed to show the association of the expression rate with prognosis using the SPSS software (Version 16, Icn, Chicago, Illinois, USA).

## Results

### CD147 expression in primary uveal melanoma

**Fig. 2:**
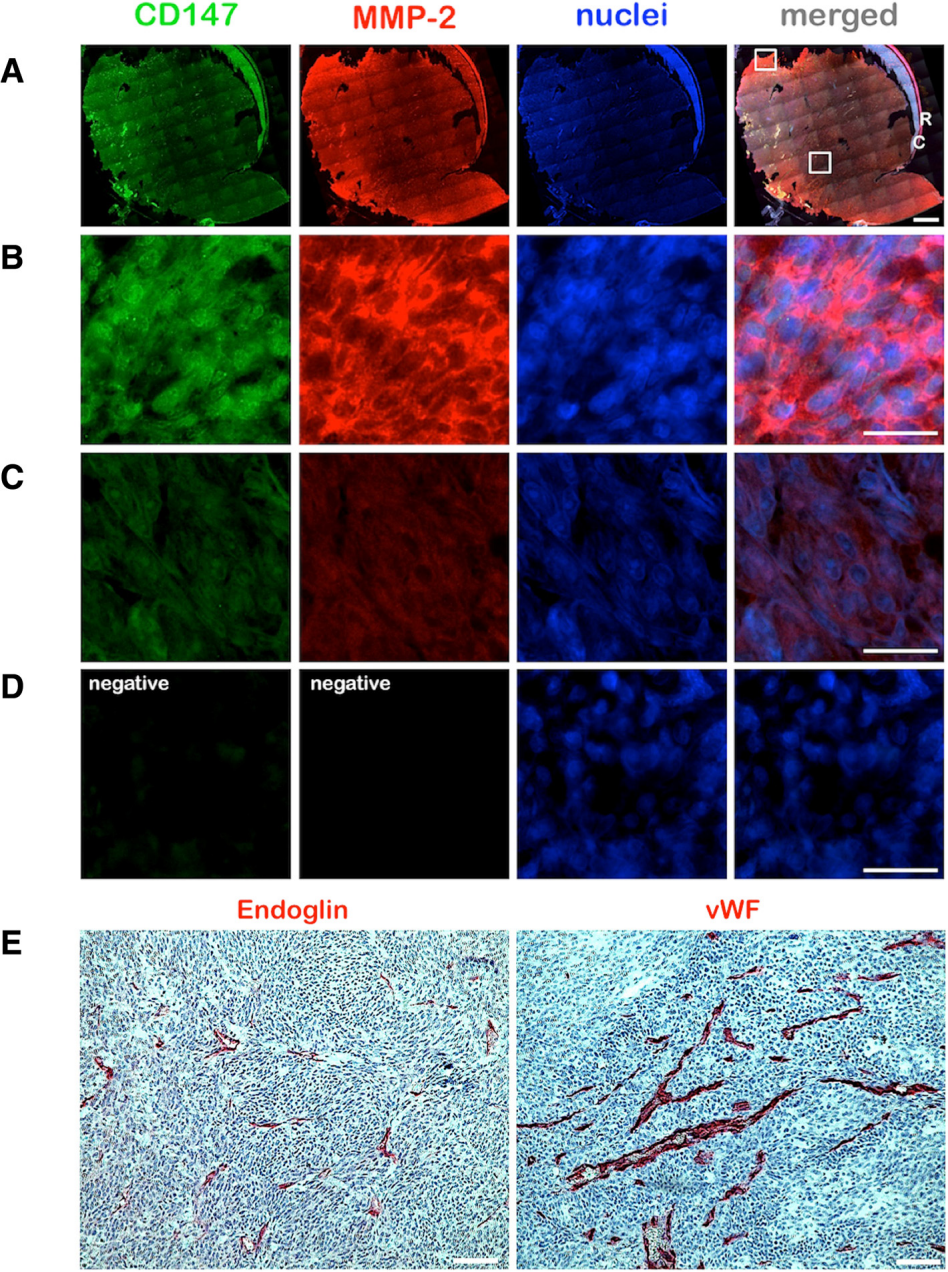
Expression of CD147 and MMP-2 in a primary uveal melanoma sample analyzed by fluorescence double-immunohistochemistry. **a** A mosaic image was constructed from *n =* 96 images acquired at 50X magnification to demonstrate the expression of CD147 (green) and MMP-2 (red) in the whole tumor (67 year old female patient who developed metastasis after 6 years of follow-up). The nuclei were counterstained with DAPI. The upper white square on the merged image demonstrates the approximate location of the images which are presented in panels B and D. The lower white square demonstrates the approximate location of the images presented in panel C. R: Retina, C: Choroid. Scale bar: 2 mm. **b**, **c** Focused images acquired at 400X magnification, which demonstrate a strong expression of CD147 and MMP-2 at the tumor margin, and a weaker expression in the inner regions, respectively. Scale bars: 25 μm. **d** Images of the negative control which was performed by omitting the primary polyclonal CD147 and MMP-2 antibodies. Scale bars (**a**-**d**): 25 μm. **e** Expression of Endoglin and vWF in the same tumor sample. Scale bars: 100 μm

**Fig. 3:**
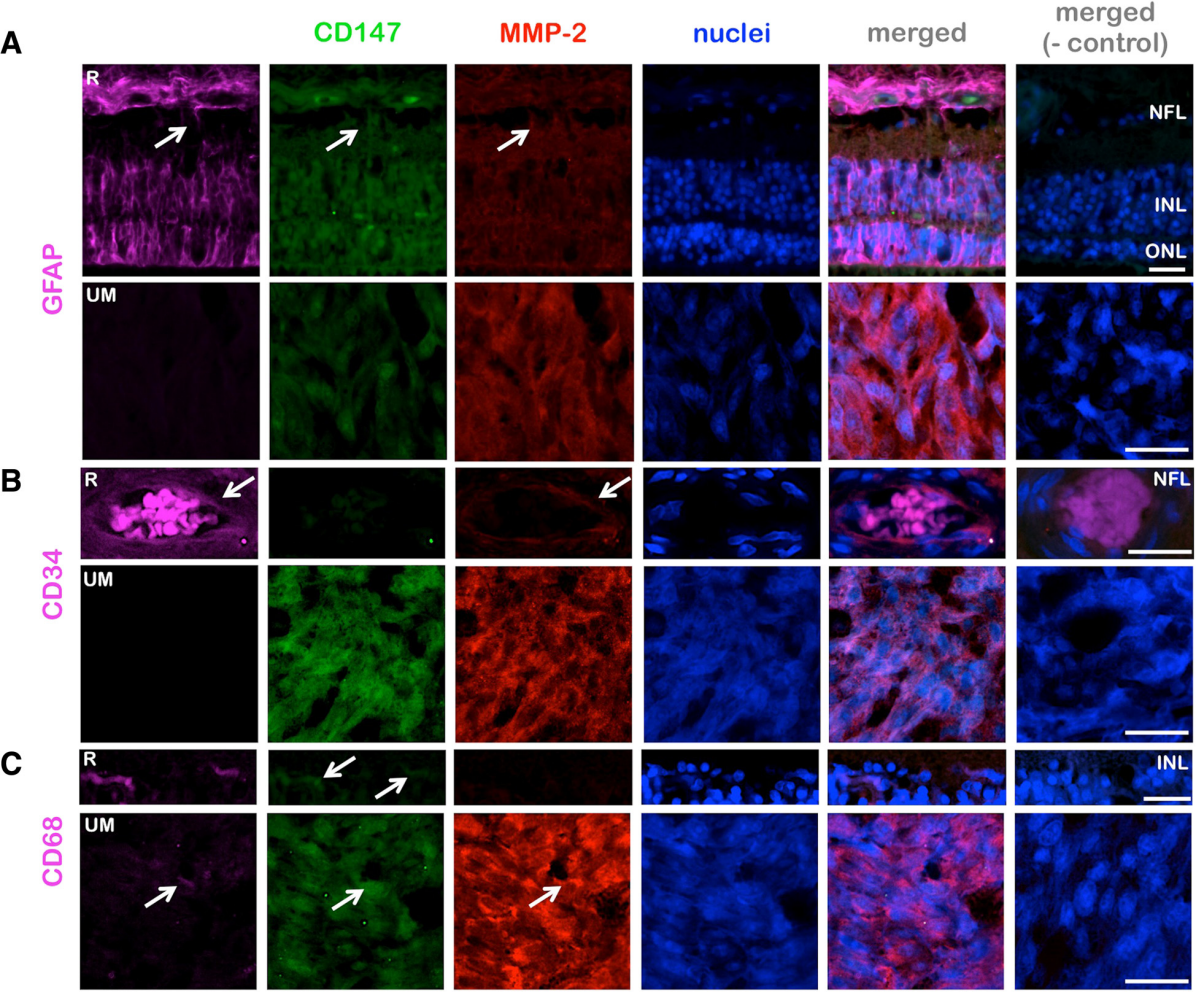
Origins of the cells exhibiting CD147 and MMP-2 expression in the uveal melanoma and retina. **a** Triple immunostainings for CD147, MMP-2, and the glial marker GFAP demonstrate the expression of the former two proteins in some astrocytes in the nerve fiber layer (NFL) and Müller cells (arrows) in the detached retina (R) above the tumor, whereas no GFAP positive cells were detected in the tumor (UM). **b** Triple immunostainings for CD147, MMP-2, and the endothelial marker CD34 demonstrate a strong expression of MMP-2 in some endothelial cells (arrows) in the detached retina (R). **c** Triple immunostainings for CD147, MMP-2 and the macrophage/fibroblast marker CD68 demonstrate the strong expression of CD147 in some microglia (arrows) in the detached retina (R). Some CD68-positive cells, which were likely to be tumor infiltrating macrophages, also exhibited a coexpression of CD147 and MMP-2 in the tumor (UM, arrows). ONL: Outer nuclear layer, INL: Inner nuclear layer. The negative (−) control was performed by the omission of all the three primary antibodies within a group. Scale bars: 25 μm

In this tumor series CD147 was expressed by 47 (96.0 %) primary uveal melanomas especially at the cell surface. The percentage fraction of positive cells according to the objective cell-count varied between 1.07 % and 100 % (mean: 37.12 %). Particularly in large tumors (LTD > 12 mm), most cells at the tumor margin exhibited a higher degree of expression compared to the cells in the inner regions (Fig. 2). In the primary tumor a weak colocalisation of CD147 and MMP-2 was observed in some CD68 positive cells, which were probably tumor-infiltrating macrophages (Fig. 3c). There was no significant difference according to the overall objective or subjective expression of CD147 in the uveal melanomas which did or did not develop metastatic disease in the further clinical course. The complete data and p-values of the objective evaluation are summarized in Table 3. The correlation with known prognostic factors (age, gender, radiation, cell type, invasion of the ciliary body, largest tumor diameter (LTD), height, TNM-classification, invasion of Bruch’s membrane, intrascleral invasion, extrascleral extension, invasion of the optic nerve) revealed no significant association with the overall expression analysis (Pearson correlation, *p <* 0.05).

**Table 3:** Comparison of objective CD147-expression of primary uveal melanoma regarding the development of metastasis

	CD147-expression of all melanomas	CD147-expression of metastatic melanoma	CD147- expression of non- metastatic melanoma	*p*-value (Mann–Whitney-*U*-test)
Objective number of positive cells (stage 1–4) mean ± SD	3.6 ± 0.74	1.4 ± 1.03	3.8 ± 0.34	
Objective intensity (stage 1–4) mean ± SD	2.8 ± 0.64	3.6 ± 0.92	1.82 ± 0.95	0.788
Rate of objective expression (%) mean ± SD	37.1 ± 25.61 %	32.6 ± 26.05 %	43.2 ± 24.28 %	0.777

*SD* standard deviation

**Fig. 4:**
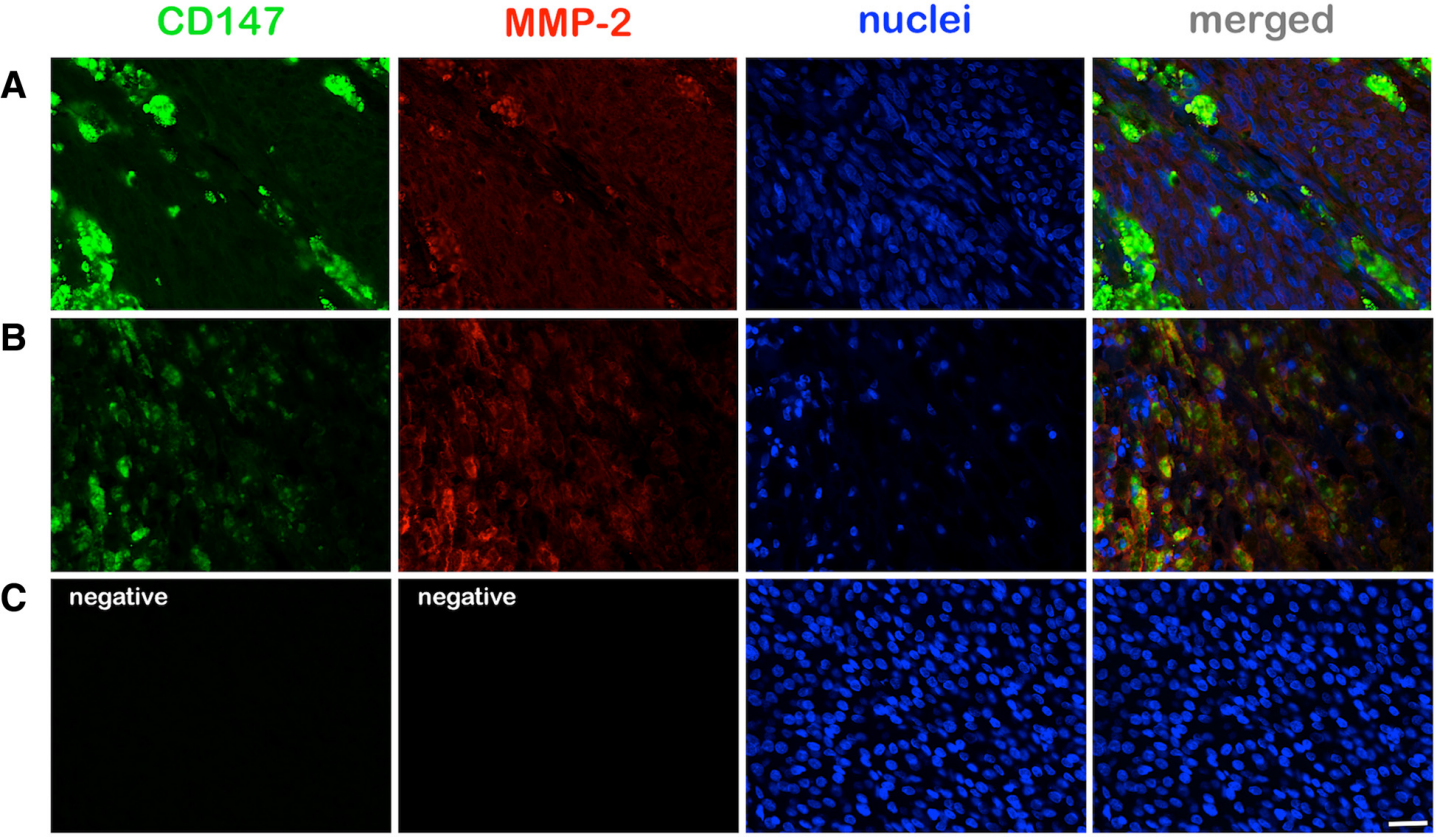
Immunofluorescence microphotography (400x magnification field of view) of (**a**) a spindle cell melanoma of a 69 year old female patient (LTD: 5 mm) revealed clustered CD147 positive cells which were associated with MMP-2 up-regulation. No metastasic disease was detected clinically during a follow-up of 9 years. In a mixed cell uveal melanoma (**b**) nested CD147 positive cells were detected which were also associated with MMP-2 upregulation. This 90 year old patient presented with metastastic disease one year after enucleation. **c** No signals were detected in the negative controls performed by omitting the primary polyclonal CD147 and MMP-2 antibodies. Scale bar: 25 μm

A closer look at the distribution of CD147 expression at higher magnification (400X) revealed that the majority of positive cells had a homogenous distribution and intensity within randomly selected fields (0.04 mm^2^, Fig. 2b and c). However, in 29 uveal melanomas (59.2 %) nested neoplastic cells were highly positive for CD147. The vast majority of these tumors with clustered CD147 positive cells (*n =* 24) developed metastasis in the further clinical course but the level of significance was not reached (*p =* 0.145) (Fig. 4). Nevertheless, in the non-metastatic sub-group, a significant correlation of clustered CD147 positive cells with LTD (*p =* 0.039), height (*p =* 0.047) and TNM-stage (*p =* 0.013) was evident. In the metastatic sub-group the presence of nested CD147 positive cells was correlated with ciliary body involvement (*p =* 0.042).

### CD147 expression in the eye

**Fig. 5:**
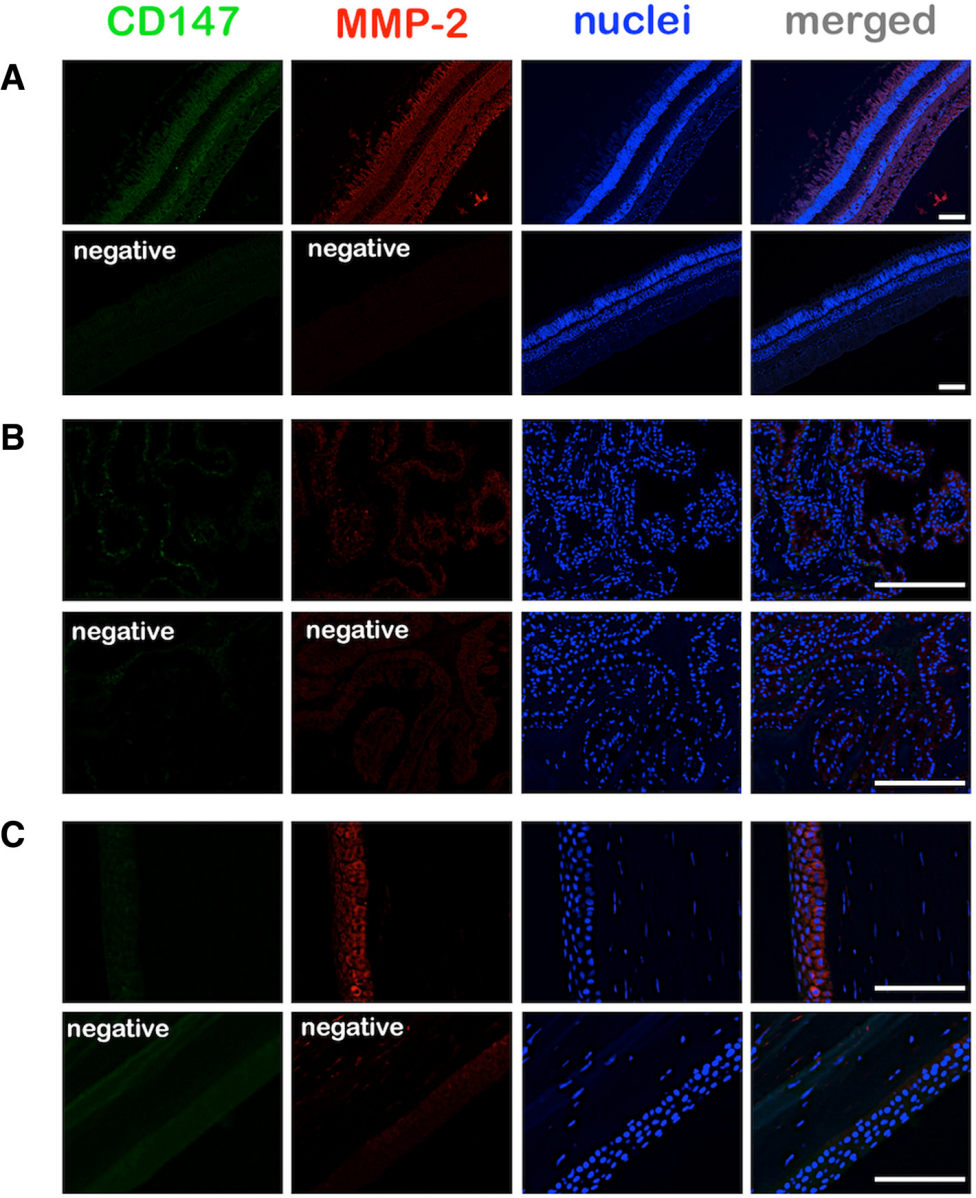
Immunofluorescence microphotography (100x magnification field of view) of the retina (**a**) revealed an MMP-2 upregulation in all retinal layers with a lower density in the outer plexiform layer. CD147 was especially upregulated in the outer and inner nuclear layer. The epithelium of the ciliary body (**b**) revealed a moderate MMP-2 expression especially at the cell surface while CD147 was expressed in a low manner. The negative controls were performed by omitting the polyclonal primary CD147 and MMP-2 antibodies. Corneal epithelium revealed CD147 positivity in contrast to the stroma of the cornea (**c**). Scale bars: 100 μm

In 35 % of the examined eyes a low or moderate CD147 expression was observed in the retina especially in the nuclear layers (Fig. 5). Coimmunostainings for CD147 and GFAP or CD68 demonstrated that some astrocytes in the nerve fiber layer, Müller cells, and CD68-positive microglia were also expressing CD147 in the retina, whereas the retinal endothelial cells (detected by antibodies against CD34) exhibited a weaker CD147 expression (Fig. 5). The epithelium of the ciliary body expressed CD147 in 15 % of the cases. The corneal endothelium and epithelium revealed a very low CD147 expression in 21 % of the documented cases. The remaining cases expressed no CD147 in the above mentioned ocular structures (Fig. 5).

### MMP-2 expression in primary uveal melanoma

MMP-2 was expressed by 47 of the 49 examined uveal melanomas in this study (96.0 %). An average of 89.3 % tumor cells showed MMP-2 expression (range: 3.26–100 %). In some large tumors (LTD > 12), cells at the tumor margin exhibited a higher degree of immunoreactivity compared to the cells in the inner regions (Fig. 1). A difference in the MMP-2 expression of tumors with or without metastatic disease could not be shown (Table 4).

**Table 4:** Comparison of objective MMP2-expression of primary uveal melanoma regarding the development of metastasis

	MMP2- expression of all melanomas	MMP2- expression of metastatic melanoma	MMP2- expression of non- metastatic melanoma	*p*-value (Mann–Whitney-*U*-Test)
Objective number of positive cells (stage 1–4) mean ± SD	3.6 ± 0.89	3.7 ± 0.82	3.6 ± 1.00	
Objective intensity (stage 1–4) mean ± SD	1.3 ± 0.87	1.2 ± 0.86	1.5 ± 0.87	0.292
Rate of objective expression (%) mean ± SD	31.0 ± 21.93 %	27.6 ± 21.01 %	35.4 ± 22.84 %	0.585

*SD* standard deviation

The correlation analysis of MMP-2 expression with known prognostic factors (age, gender, radiation, cell type, invasion of the ciliary body, largest tumor diameter, tumor height, TNM-classification, invasion of Bruch’s membrane, intrascleral invasion, extrascleral extension, invasion of the optic nerve) revealed no significant association (Pearson-correlation, *p <* 0.05). Although an increased MMP-2 expression was observed in tumors which formed VM the level of significance was not reached (*p =* 0.073).

In the detailed analysis of the distribution of MMP-2 positive cells, an up-regulation was observed in the area of the nested uveal melanoma cells which were highly positive for CD147 (Fig. 4). Beside the overall expression analysis revealed a positive correlation of those two markers (*p <* 0.001).

### MMP-2 expression in the eye

A low MMP-2 positivity was evident in some cases of the documented corneal endothelium (40 %) and epithelium (29 %). The remaining cases expressed no MMP-2. In 69 % of the examined eyes a low or moderate MMP-2 expression was observed in the retinal pigment epithelium and the detached retina associated with the uveal melanoma (Fig. 5). The retinal staining was evident particularly in the nuclear layers but also in some astrocytes, Müller cells, and retinal endothelial cells (Fig. 5). The epithelium of the ciliary body expressed MMP-2 in 40 % of the cases (Fig. 5).

## Discussion

Tumor cell invasion and metastasis formation include the ability of tumor cells to dissolute the extracellular matrix and migrate through the digested barrier. The mechanism of degradation of the connective tissues by uveal melanoma cells is poorly understood but is likely to be mediated by a variety of enzymes, including matrix metalloproteinases (MMPs) and are possibly regulated by CD147 which induces MMPs expression. CD147 is over-expressed in various cancerous tissues [9, 11, 20]. In cutaneous melanoma tissue Kanekura et al. were able to show that CD147 expression correlates with advanced tumor stage or size and is associated with a poor prognosis [15].

So far the expression of CD147 has not been studied in uveal melanomas. This study is the first to report on the expression profile of CD147 in metastatic and non-metastatic uveal melanomas. CD147 expression was observed in 47 (96.0 %) of the examined uveal melanomas. In average 37.12 % of the uveal melanoma cells were positive for CD147 (1.1–99 %). No significant association with the formation of metastasis was observed in the overall expression analysis of CD147.

Van den Oord et al. described in a series of malignant and non-malignant cutaneous lesions that MMP and CD147 immunoreactive cells were strikingly nested and alternated with unstained neoplastic cells [21]. This underlines the phenotypical heterogeneity among cutaneous melanoma cells within the same stage of tumor progression [22].

In view of these findings we screened the uveal melanoma regarding the presence of nested positive uveal melanoma cells. The presence of clustered CD147 positive uveal melanoma cells was more often observed in metastatic uveal melanomas, but the level of significance was not reached. Nevertheless, a sub-group analysis revealed a significant correlation of clustered CD147 positive cells with prognostic factors such as either LTD, tumor height, TNM-stage in the non-metastatic group or ciliary body involvement in the metastatic group.

Previous studies reported that CD147 expression level was not related to the age of the cancer patient, tumor type or gross morphology. But Zhang et al. reported on a positive correlation of CD147 expression with tumor histopathologic type and clinical stage of disease in a series of squamous cell carcinoma [23, 24] as observed in our series of uveal melanomas.

Nevertheless the exact mechanism of metastasis formation and the exact impact of CD147 is still unknown. In our study nested uveal melanoma cells expressed CD147 especially at the cell surface. CD147 seems to require direct cell-cell contacts to stimulate collagenolytic activity, CD147 on melanoma cells might act on neighbouring neoplastic cells to induce the release of enzymes necessary for the breakdown of matrix components as already proposed by van den Oord et al. based on their observation in pigmented skin lesions [21]. Additionally, we observed an up-regulation of CD147 and MMP-2 associated with CD68 positive cells as shown previously for other tumours. These results indicate a mechanism whereby tumour cell-macrophage interactions induce the EMMPRIN-MMP pathway as supposed by Amit-Cohen et al. [25].

Accordingly, our findings confirm that an increased CD147 expression was significantly associated with an increased MMP-2 expression. This relationship was described for different tumors but not for the uveal melanoma so far. Previous studies demonstrated that CD147 is involved in regulation of tumour progression including growth, invasion and metastasis formation via induction of MMPs secretion from surrounding fibroblasts [26, 27]. Wysocki reported that CD147 which was purified from hepatocellular cancer (HCC) cells stimulates human fibroblasts to produce MMP-2 and MMP-9 [28]. HCC cells with silenced MMP-2 and MMP-9 genes were less invasive compared to MMP-2 and MMP-9 silenced fibroblasts or CD147-silenced HCC cells when co-cultured with fibroblasts [26, 29]. The use of siRNA, a specific antibody, or arsenic trioxide to block CD147, decreases the MMP-2 and MMP-9 secretion resulting in an inhibition of migration and invasion of HCC [26, 30]. Therefore, the presumed regulative relationship of these two proteins might also apply to uveal melanomas.

The CD147 induced up-regulation seems to be a key event during *vasculogenic mimicry* formation in tumors as demonstrated in a previous study for ovarian cancer cells [31]. According to these findings we also observed a non-significant up-regulation of MMP-2 in *VM* positive uveal melanomas, which may indicate a prominent relevance of MMP-2 for *VM*. Besides, due to its protease function MMP-2 has a crucial role during the degradation of extracellular matrix (ECM) which results in the reduction of adhesive strength between tumor cells, induction of migration, and potentially metastasis formation [32, 33]. An increase of MMPs expression was observed in a number of tumors during progression. Especially higher MMP-2 and MMP-9 expression was correlated with invasive and metastatic tumor properties [34, 35]. A number of different tumors such as of the colorectum [36], breast [37], lung [38], liver [39], skin [40], prostate [41], and ovary [42] reveal an elevated MMP-2 level. Cottam et al. reported on an increased MMP-2 secretion in different uveal melanoma cell lines [43]. Additionally, findings of an in vitro study indicate that the activated MMP-2 expression may be associated with uveal melanoma progression [44]. An immunohistochemical analyse of 29 uveal melanoma specimens revealed an association of an increased MMP-2 expression with a worse prognosis [45]. In our series of uveal melanomas no significant association of MMP-2 expression and prognosis was evident.

CD147 is proposed not only to be involved in *vasculogenic mimicry* formation but also in the regulation of angiogenesis via VEGFR-2. Tang et al. have recently reported that the up-regulation of EMMPRIN in MDA-MB231 breast tumor cells can also increase VEGF expression in these cells, which can then act in a paracrine manner on endothelial cells to promote tumor angiogenesis. Similarly, EMMPRIN seems to promote melanoma cell invasion and disease progression by stimulating the VEGF/VEGFR-2 autocrine loop in two cutaneous melanoma cell models [46]. Hypoxia itself may enhance the CD147 induced MMP-2 expression, the invasive and metastatic potential of cutaneous melanoma cells as presumed by Bougatef et al. [47]. Besides Desch et al. postulate that MMP-2 itself apparently plays a central role in the autocrine regulation of VEGF-A secretion at the transcription level and expression which directly targets VEGFR-2 leading to endothelial cell activation in cutaneous melanoma cell lines [48]. In our series of 49 uveal melanomas no significant association of CD147 or MMP-2 expression with tumor associated angiogenesis by means of Endoglin expression or the vWF expression itself was observed. This might be due to the advanced stage of evaluated uveal melanoma tissues with a mean size of 15.5 mm. In these cases the rapid growth phase and therefore the process of tumor induced neovascularisation might be already completed which is a hypothesis already proposed for cutaneous melanoma [49].

The additional analysis of MMP-2 expression in this series of eyes with uveal melanomas revealed an upregulation of MMP-2 in the retina under ischemia or induced by reperfusion injury, oxidative stress which both may occur in the case of retinal detachment and mechanical stretching as described in previous studies [50–53] due to the tumor growth underneath.

## Conclusions

In summary, CD147 up-regulation was significantly correlated with a higher MMP-2 expression in this series of uveal melanoma. The presence of clustered CD147 in uveal melanomas was significantly associated with tumor stage in the non-metastatic subgroup.

## Abbreviations

BSA: bovine serum albumin
CD 31: cluster of differentiation
CD 34: cluster of differentiation
CD 68: cluster of differentiation
CD147: matrix-metalloproteinase inducer
CD105: Endoglin
Dapi: 4′,6-Diamidin-2-phenylindol
ECM: extracellular matrix
EMT: Epithelial-mesenchymal transition
GFAP: Glial fibrillary acidic protein
HCC: hepatocellular cancer
HNSCC: head and neck squamous cell carcinoma
IgG: immunglobuline G
JL: Julia Lüke
LTD: largest tumor diameter
MMP: matrix-metalloproteinase
PBS: phosphate balanced saline
SPSS: Statistical Product and Service Solutions
TNM: tumor node metastasis
TGF- β1: TGF-β3, transforming growth factor-β1 and –β3
*VM*: vasculogenic mimicry
vWF: von Willebrand Factor
VV: Vlatka Vukoja
